# Adherence to 6-Mercaptopurine in children and adolescents with Acute Lymphoblastic Leukemia

**DOI:** 10.1371/journal.pone.0183119

**Published:** 2017-09-06

**Authors:** Mervat Alsous, Rana Abu Farha, Eman Alefishat, Suha Al Omar, Deema Momani, Alia Gharabli, James McElnay, Robert Horne, Rawad Rihani

**Affiliations:** 1 Department of Clinical Pharmacy and Therapeutics, Faculty of Pharmacy, Applied Science Private University, Amman, Jordan; 2 Department of Biopharmaceutics and Clinical Pharmacy, Faculty of Pharmacy, the University of Jordan, Amman, Jordan; 3 Clinical Pharmacy Department, King Hussein Cancer Center, Amman, Jordan; 4 Clinical and Practice Research Group, School of Pharmacy, Queen’s University Belfast, Belfast, United Kingdom; 5 Centre for Behavioural Medicine, UCL School of Pharmacy, University College London, London, United Kingdom; 6 Department of Paediatric Oncology, Paediatric Bone Marrow and Stem Cell Transplantation, King Hussein Cancer Center, Amman, Jordan; Pennsylvania State University, UNITED STATES

## Abstract

**Objective:**

Studies on children with Acute Lymphoblastic Leukemia (ALL) reported non-adherence in 2–54% of cases. The primary objective of this study was to assess rates of adherence to 6-MP using two different methods in children and adolescents with ALL. Secondary aim was to identify factors that influence adherence to 6-MP in children with ALL.

**Methods:**

All eligible children with ALL who are (≤ 19) years old and receive 6-MP therapy for at least 1 month were approached to participate in the study. A total of 52 children with ALL and their primary caregivers were recruited. Adherence measures included an objective method (measuring 6-MP metabolites in packed Red Blood Cells (RBCs)) and a subjective method (using parent and child self-report via the Medication Adherence Report Scale; MARS; Adherence was defined as 90% or greater).

**Results:**

Rates of adherence varied across the measurement methods. Packed RBCs sample analysis indicated forty-four patients (84.6%) to be adherent. Using the MARS questionnaires, a total of 49 children (94.2%) were classified as being adherent according to the parental MARS questionnaire scores, while all the 15 children (100%) who answered the MARS (child) questionnaire were classified as adherent. Overall adherence rate was 80.8% within the studied population.

**Conclusion:**

MARS scale was shown to overestimate adherence compared to measurement of 6-MP metabolites in the blood. A combination of both methods led to increased detection of non-adherence to thiopurine in children with ALL.

## Introduction

Acute lymphoblastic leukemia (ALL) is the most common cancer in children[1]. Maintenance therapy in ALL consists of daily doses of 6-mercaptopurine (6-MP), weekly methotrexate interrupted by monthly pulses of vincristine and dexamethasone[2]. 6-MP is considered the most easily monitored treatment due to its daily administration and stable intracellular metabolites[3]. 6-MP is a drug with cytotoxic effects that has a short half-life (1.5 hours), so measuring its plasma level cannot be used to assess drug effect. However, intracellular accumulation of 6-MP metabolites [6-thioguanine (6-TGN) and 6-methyl mercaptopurine (6-mMPN)] occurs over a period of 2–3 weeks; measuring the concentration of the metabolites is essential in therapeutic drug monitoring and considered as a good indicator of long term adherence[4]. 6-TGN is considered the most active thiopurine metabolite[5], while 6-mMPN is considered responsible for the side-effects of thiopurine therapy[6]. Measuring 6-MP metabolite levels in blood or urine provide an objective measure of adherence to medication; however, this methodology can be expensive and is not always available [3]

Poor adherence to medication treatment is a recognised problem in paediatric patients, where non-adherence rates are reported to range from 25% to 60% [7–10]. Since paediatric ALL patients become practically asymptomatic once remission is attained, but continue to require complex treatment, non-adherence to prescribed treatment in ALL patients is expected[11]. Studies in children and adolescents with ALL reported non-adherence to 6-MP in 17–46% of the cases [3,12–14]. This problem has several negative consequences such as repeated clinic visits, extended course of illness, poorly controlled symptoms and increased cost due, for example, to unnecessary hospital admissions[15]. Therefore, non-adherence is a significant concern in all illnesses especially in patients with chronic conditions, including children.

There is no standard limit for what constitutes adequate adherence, with some trials considering adherence rates > 95% as being essential. [16]. Different methods of adherence assessment have been reported including prescription refills, pill counts, electronic monitors built into medication containers, blood levels of the drug, patient self-report and physician evaluation of patient adherence [17,18]. However, there are no methods without methodological limitations[19].

Reports on non-adherence to oral 6-MP have relied on small cohorts of patients and been based largely on self-report [13,20–23]. However, this approach can be susceptible to misrepresentation and may overestimate a patient's adherence [17,18,24]. Using cutt-off point limits for assessing adherence to 6-MP (using blood levels) is susceptible to bias due to high variability of drug concentration in patients even if they receive the same dose. Therefore, hierarchical cluster analysis of drug metabolite concentrations (which was used in this study) is a good approach as an objective method to assess non-adherence to 6-MP [3]. The use a multi-method approach (at least two adherence measurement sources) for assessing adherence is recommended to increase reliability of detecting non-adherence [3].

The rate of non-adherence to 6- MP is reliably unknown in children with ALL. This study is the first to evaluate adherence to 6-MP using two measurement methods in Jordan (blood level determinations using hierarchical cluster analysis, and via patient and parent self-report). Secondary aim was to identify factors that influence adherence to 6-MP in children with ALL

## Methods

### 2.1 Study subjects, setting and data collection

The study was approved by the research Committees (IRB) in King Hussein Cancer Centre (KHCC), Amman-Jordan (Reference number: 15KHCC 57). Recruitment of study subjects occurred over the period from September, 2015 to September, 2016. Informed parental consent was obtained for each child before enrolment in the study. In addition, verbal assent was obtained from older children (>6 years) after provision of a verbal description of the study and what it involved. All eligible patients and their parent were approached (n = 53) and only one parent refused to participate with his child in the study. Data were collected prospectively from paediatric patients (≤19 years) (n = 52) attending the Outpatient Paediatric Leukemia Clinic at KHCC and who had been receiving continuous/maintenance 6-MP therapy for at least 1 month. Children at KHCC are treated based on the St. Jude total XV and total XIII protocols[25,26]. According to this approach, the continuation phase consists of 120 weeks for females and 146 weeks for males. In this phase, patients receive different chemotherapeutic agents with different dosing regimens including 6-MP, high dose and low dose methotrexate, vincristine, dexamethasone and asparaginase depending on the risk category of the patient. Post week 24 of the continuation phase, all patients are maintained on the same dose of 6-MP (75mg/m2/day) daily until the end of treatment. The dose of 6-MP is modified (increased or decreased) if the patient has a persistently high acute neutrophil count or if the patient has recurrent toxicities (myelosuppression) respectively. 6-MP is administered in the evening on an empty stomach. Patients who had appointments in the leukemia clinic were identified, and were considered for study inclusion if they were in the continuation phase post week 24 of treatment and if they had been maintained on 6-MP for at least 1 month. If 6-MP was stopped for more than 3 days, patients were excluded.

Once a patient was recruited, data were obtained from their clinical files, which included demographic data, medical history, biochemical data (complete blood count and liver function tests), and information relating to side-effects of 6-MP (elevated liver enzymes, neutropenia).

Whole blood samples (one sample per patient; 2 mL aliquot) were taken from a routine clinical blood sample withdrawn from the child at the clinic. All the blood samples were labeled with the patient study number together with the date and time of collection. Blood samples were collected by a research assistant and processed within 4 hours of taking the sample.

The Arabic translated and validated version of Medication Adherence Report Scale (MARS) [27] was completed by parents and by older children (≥ 11 years). The parent's version consisted of six questions; mean scores were summed to give a scale score ranging from 1 to 5. The child's version consisted of five items; mean scores were summed to give a scale score ranging from 1 to 5. Higher scores indicate higher levels of self-reported adherence. In the present study, 90% cut-off points for adherence were used, i.e. a participant was considered to be adherent, if the parental/ child MARS score was at least 4.5 out of 5. This cut-off point was selected to represent situation where even relatively fluctuations in adherence may be clinically significant.

### 2.2 Analysis of blood samples

All venous blood samples obtained from patients at the clinic visit were processed into plasma and packed red blood cell (RBC) samples and stored at -80°C prior to analysis at the Pharmaceutical Research Center, Jordan university of Science and Technology.

A sensitive, selective, microanalytical method for determination of the 6-MP metabolites in packed RBCs was developed and validated (S1 Appendix). The method utilized 100 μl of packed RBCs. The sample preparation step involved the addition of 100 μl dithiothreitol (75 mg/mL) to the 100 μl of packed RBCs and 150 μl of water. The sample was vortexed for 1 minute after which 50 microlitres of perchloric acid (700 mL/L) was added and vortex-mixed for a further 30 seconds. The sample was then centrifuged at 13,000 × g for 15 minutes at 4°C, and all the clear supernatant layer was removed and heated for 45 min on 100°C using a heating block. After cooling, 700 µL of water was added and the solution vortex-mixed for 10 seconds before being transferred to MCX solid phase extraction (SPE) cartridges (1 ml/30 g; Waters). The sample mixture was then dried under a stream of nitrogen at 37°C for 20 min and reconstituted in 100 µL 0.05 M NaOH with vortex-mixing for 1 minute. Samples were analyzed using high-performance liquid chromatography (HPLC) with UV detection (322nm and at 342 nm). The assay limits of quantification for 6-TGNs and 6-mMPNs were 0.5 and 3.75 μM, respectively. All inter- and intra-day accuracy and precision measures obtained were satisfactory according to the Food and Drug Administration guideline (±15%) [28] ranged from −7.0% to 12.62%. Concentration ranges covered by the assay validation were 0.5–20μM for 6-TGNs and 3.75–300μM for 6-mMPNs.

### 2.3 Study measures and adherence assessment

Adherence to 6-MP was assessed using levels of 6-MP metabolites in packed RBCs and results from parent and child self-report using the MARS questionnaires (Table 1).

**Table 1:** Classification of medication-adherence using different measures.

Adherence measure	Type of measure	Adherent	Non-adherent
**TGNs and 6-mMPs levels in packed RBCs**	Objective	Concentrations of both 6-TGN and 6-mMP above a threshold value*	Concentrations of both 6-TGN and 6-mMP below a threshold value
MARS**(parent/caregiver)	Subjective	If MARS mean score is ≥ 4.5	If MARS mean score is <4.5
**MARS (child)**	Subjective	If MARS score is ≥ 4.5	If MARS mean score is <4.5

* (20^th^ percentile) of 6-TGNs and 6-mMPNs levels

** MARS: Medication Adherence Report Scale

The correlation between the concentrations of both metabolites was studied [3,29]. Patients were clustered into four categories according to their 6-TG and 6-mMPNs levels. Cluster A (i.e. above 20^th^ percentile cutoff point) was characterized by high levels of 6-TGN levels and 6-mMP levels (positive correlation) thus patients were considered adherent to medication. Cluster B was characterized by negative correlations between the two metabolites (high levels of 6-mMP but with low 6-TGN concentrations), it is expected that those patients had higher TPMT activity which shift 6-MP metabolism toward 6-mMP production. Cluster C was characterized by low levels of both 6-mMP levels and 6-TGN (non-adherent patients, i.e. less than 20^th^ percentile cutoff point). Cluster D patients have also (negative correlations between the two metabolites) low levels of 6-mMP levels and high levels of 6-TGN, it is expected that those patients had lower TPMT activity which shift 6-MP metabolism toward 6-TG production.

### 2.4 Data analysis

Descriptive statistics (mean and standard deviation (SD) for continuous data, number (N) and percentage (%) for categorical data) were used to describe participants’ demographic characteristics, disease characteristics and adherence. If continuous variables were not normally distributed, median and range were used. Cluster analysis, using the 20th percentile as a cutoff point, was used to investigate the pattern of 6-TGN and 6-mMP metabolite concentrations in patients with ALL and to group these patients according to their metabolite levels.

Factors that were linked to adherence to 6-MP treatment were identified using the data obtained from the patients' medical notes and demographic characteristics. Group differences (adherent and non-adherent) were explored using Mann Whitney U test for continuous variables. Categorical variables were analyzed using Chi-squared analysis. If the expected frequency fell below 5, the Fisher’s exact test was employed. All analyses were carried out using SPSS^®^ software version 22. The significance level was set at 0.05.

## Results

### 3.1 Patients and disease characteristics

Fifty-two eligible patients and their primary caregivers/parents (n = 52) were approached and agreed to take part in this cross-sectional study. The demographics and disease characteristics of the study sample are described in Table 2. Thirty-one patients were male (59.6%) with the mean age of 8.9 years (SD = 4.4). On the other hand, 10 parents were males (19.2%) with the mean age of 36.2 years (SD = 7.4).

**Table 2:** Demographic and disease characteristics of the study sample (n = 52)*.

Parameters	Results
**Patient's age (years)**	8.9 (4.4)
**Patient's gender, n(%)** ▪ Males ▪ Females	31 (59.6) 21 (40.4)
**Patient's weight (kg)**	33.4 (20.8)
^**^Parents age (years)	36.2 (7.4)
^**^Parents gender, n (%) ▪ Males ▪ Females	10 (19.2) 42 (80.2)
**Disease duration(years)**	1.73 (1.61)
**6-MP daily dose (mg/m2), median (range)**	66.59 (44.6–107.9)
**6-MP daily dose (mg/kg), median (range)**	2.23 (1.05–3.18)
**Metabolite concentrations**	
6-TGNs (μM), median (range)	4.2 (undetectable–10.6)
6-mMPNs (μM), median (range)	52.58 (undetectable–123.9)
**Number of medications, n (%)** ▪ 2 ▪ 3 ▪ 4 ▪ ≥5	5 (9.6) 23 (44.2) 14 (26.9) 10 (19.2)
**Presence of side effects, n (%)** ▪ Yes	11 (21.2)
**Type of side effect, n (%)** ▪ Decrease ANC ▪ Elevation ANC ▪ Hepatic toxicity ▪ Neutropenia ▪ Severe MTX neurotoxicity ▪ Hyperbilirubinemia ▪ Hypersensitivity to 6-MP	2 (3.8) 3 (5.8) 1 (1.9) 2 (3.8) 1 (1.9) 1 (1.9) 1 (1.9)

*Data presented as mean (SD) unless otherwise stated

^**^ Parents who filled out the MARS questionnaire.

SD: Standard deviation

ANC: Absolute neutrophil count

MTX: Methotrexate

The mean duration of illness was 1.73 years (SD = 1.61). The number of medications received by patients ranged from 2–7 medications (median = 3) [Data was not normally distributed].

### 3.2 Adherence assessment

#### 3.2.1 6-MP metabolite concentrations in plasma and packed RBC samples

**Fig 1:**
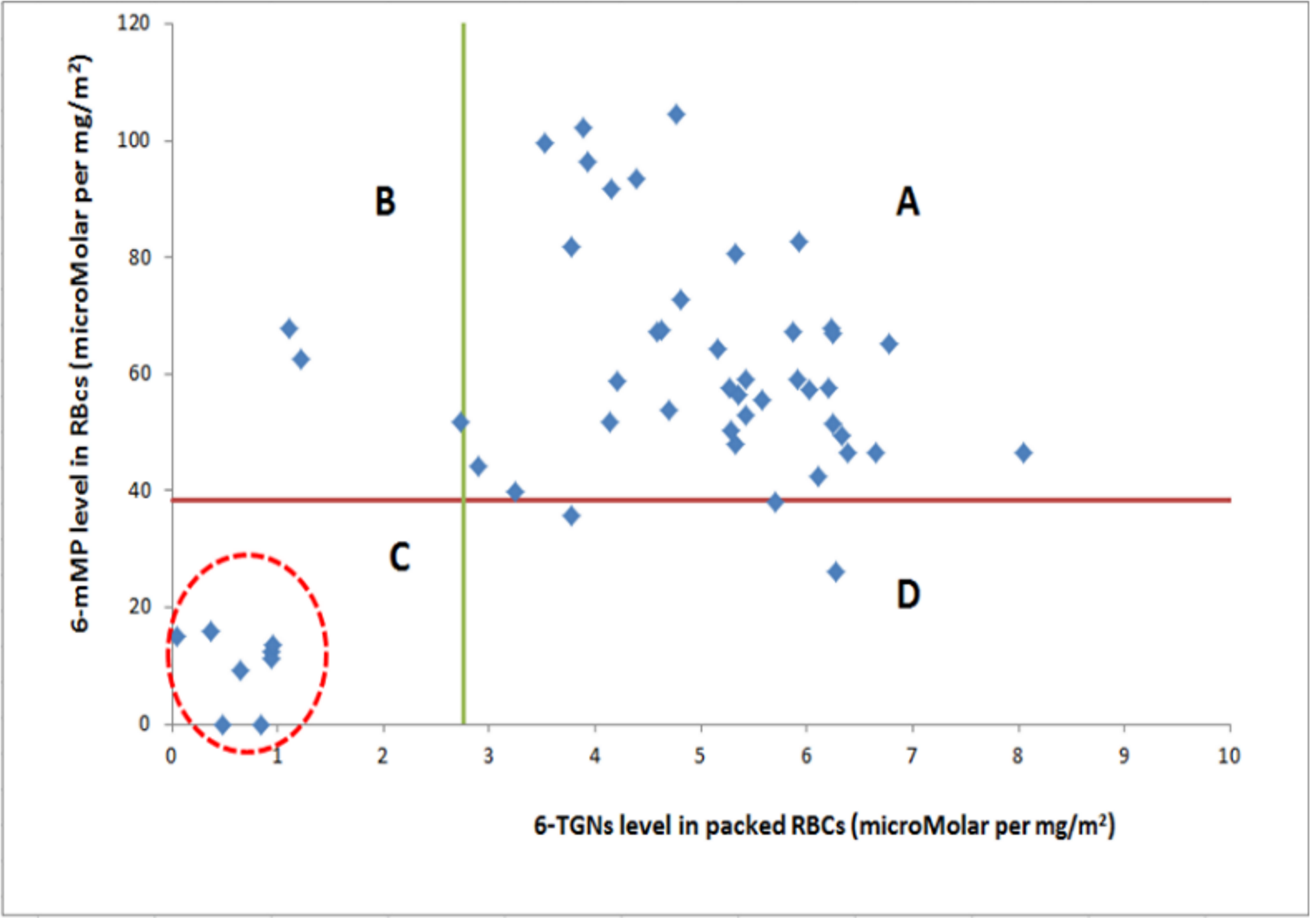
Scatter plot showing the four different clusters formed after hierarchical clustering of the ALL study sample using 20th percentile of metabolite levels as cutoff point. Data for 6-mMPNs and 6-TGNs are the metabolite levels (adjusted per dose/SA). Cluster A (i.e. above 20% cutoff point) was characterized by high levels of 6-TGN levels and 6-mMP levels (adherent patients). Cluster B was characterized by high levels of 6-mMP but with low 6-TGN concentrations, it is expected that those patients in Cluster B had higher TPMT activity than those in cluster A, which would explain the shift in 6-MP metabolism toward 6-mMP production in those patients. Cluster C was characterized by low levels of both 6-mMP levels and 6-TGN (non-adherent patients). Cluster D patients have low levels of 6-mMP levels and high levels of 6-TGN (Lower TPMT activity in patients in Cluster D compared to those in cluster A would explain the shift in 6-MP metabolism toward 6-TG production in those patients).

A total of 52 venous blood samples (processed into packed RBCs) were obtained from the 52 study patients. Both 6-TGN and 6-mMP metabolite concentrations were measured for all study subjects. Based on cluster analysis [3], patients were segregated according to their adherence; forty-four patients (n = 44, 84.6%) were considered adherent and 8 children were classified as non-adherent (15.4%) Fig 1.

#### 3.2.2 Adherence using MARS-specific (parent and child versions)

Score distribution for the MARS questionnaires is presented in Table 3. Children were classified as being adherent if the total score was greater than or equal to the 90^th^ percentile of the maximum score (i.e ≥ 4.5 for MARS (parent and child)). Accordingly, using the parental total MARS questionnaire scores, a total of 49 children (94.2%) were classified as being adherent. On the other hand, among the 15 children who answered the MARS (child) questionnaire (those aged 11 years or above), all children (100%) were classified as being adherent.

**Table 3:** Distribution of the total scores for the MARS questionnaires reported by participating parents and children.

Measure	n	Total score mean	Total score range	Number (%) of non-adherent patients (score < 4.5)
MARS^*^ **(Parent)**	52	4.8	4.3–5	3 (5.8%)
MARS^*^ **(Child)**	15	4.9	4.6–5	0(0%)

* MARS: Medication Adherence Report Scale

### 3.3 Comparison between different methods of adherence assessment

Packed RBCs sample analysis captured the highest percentage of non-adherence (15.4%) followed by the MARS (parent) questionnaire (5.8%). MARS (child) questionnaire did not identify any non-adherence (0.0%). Combination of these assessment approaches (classified as non-adherent if either method identified patient as non-adherent) resulted in an overall non-adherence rate of 19.2%.

The level of agreement between the packed RBCs and the MARS (parent) methods was found to have Kappa = 0.107 (p-value = 0.375, 95% CI = -0.204–0.438).

### 3.4 Factors affecting overall adherence to 6-MP

An investigation of the factors affecting adherence to 6-MP in children with ALL was performed. None of the studied factors (child age, parent age, child gender, parent gender, parent educational level, duration of ALL treatment, number of medications, and the presence of side effects) were found to affect adherence (p>0.05), therefore a multivariate analysis was not conducted.

## Discussion

This is the first study in Jordan that investigated the adherence to 6-MP in children and adolescents with ALL utilizing an objective method (measuring 6-MP metabolites in packed RBCs) as well as a subjective method (self-reported questionnaires) to determine non-adherence to 6-MP treatment. Since non-adherence is potentially life threatening in children with ALL, a multi-method approach for assessing adherence is an appropriate way of increasing sensitivity and reliability in detecting non-adherence[3].

Adherence to prescribed treatment is a complex and multi-factorial matter that is poorly understood[30]. It is a key element for the evaluation of treatment efficacy and safety, thus adherence plays an important role in clinical research and practice. The use of different ways in assessing adherence decreases bias associated with using a single method[31–33].

The Arabic version of the MARS-specific questionnaire [27] was the self-report questionnaire used for adherence assessment in the present study. The majority (94.2%) of parents of ALL patients reported that their children were adherent to their 6-MP treatment, while none of the patients reported being non-adherent to 6-MP. This is consistent with the reported overestimation of adherence using this method [34–37].

The objective measure utilized in the present study included clustering patients according to their 6-MP metabolite concentrations (hierarchical cluster analysis). A 20^th^ percentile cut-off point for both metabolites was used to identify non-adherence to therapy. The idea of cluster analysis regarding 6-MP active metabolites has been applied in two previous studies [3,29]. Traore *et al* used this method to identify patients at risk of suboptimal 6-MP therapy [29], while Hawwa *et al* used it as a novel approach to assess non-adherence to oral thiopurines [3]. Upon the application of the objective method, eight ALL patients (15.4%) were considered non-adherent to medication. These patients had their 6-mMPNs and 6-TGNs located within cluster C in Fig 1 which represented low or undetectable levels of the metabolites (Fig 1).

The intracellular metabolism of 6-MP depends on the activity of thiopurine-S-methyltransferase enzyme (TPMT) which is subjected to genetic polymorphism with an inverse correlation between 6-TGNs levels and TPMT activity[38]. It is reported that the concentrations of 6-MP metabolites are subjected to large inter-individual variations[39].Unfortunately, information on patient genotypes as TPMT testing is not used in the hospital. Having access to the latter information can help differentiate patients who are received suboptimal 6-MP doses (Cluster B and D). It is expected that those patients in Cluster B had higher TPMT activity than those in cluster A, which would explain the shift in 6-MP metabolism toward 6-mMP production in those patients. Lower TPMT activity in patients in Cluster D compared to those in cluster A would explain the shift in 6-MP metabolism toward 6-TG production in those patients.

The objective method (RBC metabolite concentrations) was more effective in identifying non-adherent patients (8 patients, 15.4%) than the subjective, parental self-report approach (3 patients, 5.8%) while the child self-report approach did not identify any cases of non-adherence. Interestingly, two of the three patients identified as non-adherent using parental self-report (MARS-specific) were not identified using the metabolite data. This could represent good adherence in the period coming up to the clinic attendance (acute adherence) and highlights the importance of using a multi-method approach within this challenging field. When both methods were combined a total of 10 patients were classified as non-adherent, i.e. overall non-adherence was 19.2% which is similar to the reported range in international literature [3,12–14,40–42]. In addition, those parents of non-adherent patients might not be involved in giving the medication and children were responsible for taking the medication by themselves.

None of the factors studied (including patient age, parent age, patient gender, parent gender, parent educational level, duration of ALL treatment, number of medications, and the presence of side effects) were found to be associated with patient adherence to 6-MP.

Unfortunately we were not able to identify factors affecting adherence in children patients with ALL and this may be due to the small sample size however, this is the cohort in Jordan as recruitment was conducted over one year and all eligible patients were approached and more than 98% agreed to participate (52 out of 53 patients). In addition, other drivers of non-adherence or false report of adherence (eg. sociodemographic status [22], parental beliefs about medicines (BMQ), Patient’s and/or parent’s primary reasons for skipping medication doses due to their busy schedules [43], RBC transfusions from a donor with high TPMT activity) were not assessed in this research. These issues can be addressed in future studies with inclusion of larger number of patients form different Arabic countries around Jordan.

### Limitations

The population of children with ALL investigated in the present study was relatively small, however, it is larger than many cohorts of children and adolescents published thus far. Adherence assessment was based on one blood sample for each patient; the inclusion of multiple samples obtained at various time points may have resulted in a more accurate assessment of adherence [39]. A further potential limitation of the study is that TPMT testing is not used in Jordan. A major strength of the present study was the use of both objective and subjective measures for assessing adherence as this is one of the first studies to utilize an objective method to measure adherence in ALL in Jordan.

## Conclusion

In a serious, life threatening condition like ALL in children, one might expect full adherence with curative medication. This obviously was not the case, even within healthcare system where medications are made available. The study highlights the importance of using both subjective and objective measures of adherence in identifying true adherence. The work highlights the need for more robust education and monitoring programs for children and young people with ALL, to help parents and patients better understand the disease and major benefits of good adherence. The latter should be reinforced regularly with both parents and patients.

## Supporting information

S1 Appendix Development and validation of an HPLC method for the determination of 6-MP in packed red blood cells and plasma. (PDF)
