# Supplementary material for: Adherence to 6-Mercaptopurine in children and adolescents with Acute Lymphoblastic Leukemia
Source: PLoS One. 2017 Sep 6;12(9):e0183119. doi: 10.1371/journal.pone.0183119 (PMC5587295; doi:10.1371/journal.pone.0183119)
Supplement: S1 Appendix — (PDF) [file pone.0183119.s001.pdf]

DEVELOPMENT AND VALIDATION OF AN HPLC METHOD FOR THE DETERMINATION OF AZATHIOPRINE/6-MERCAPTOPURINE METABOLITES IN PACKED RED BLOOD CELLS AND PLASMA

Introduction

1.1 HPLC

Liquid chromatography (LC) is an analytical technique that was defined by the work of the Russian botanist Mikhail S. Tswett (early 1900s). His research focused on separating a mixture of compounds from plants using a solvent and a column packed with absorbent material (Waters, 2013a).

High performance liquid chromatography (HPLC) is now one of the principal tools in analytical chemistry. It is involved in the separation and quantification of different compounds that are dissolved in a liquid phase (Rouessac and Rouessac, 2007).

High Performance Liquid Chromatography (HPLC) requires the delivery of the liquid mobile phase under high pressure (up to 350 bars) under conditions which ensure a stable flow rate and avoids pulsation even when using different compositions of the mobile phase (Snyder *et al.*, 2011). Figure 1 illustrates a typical HPLC system.

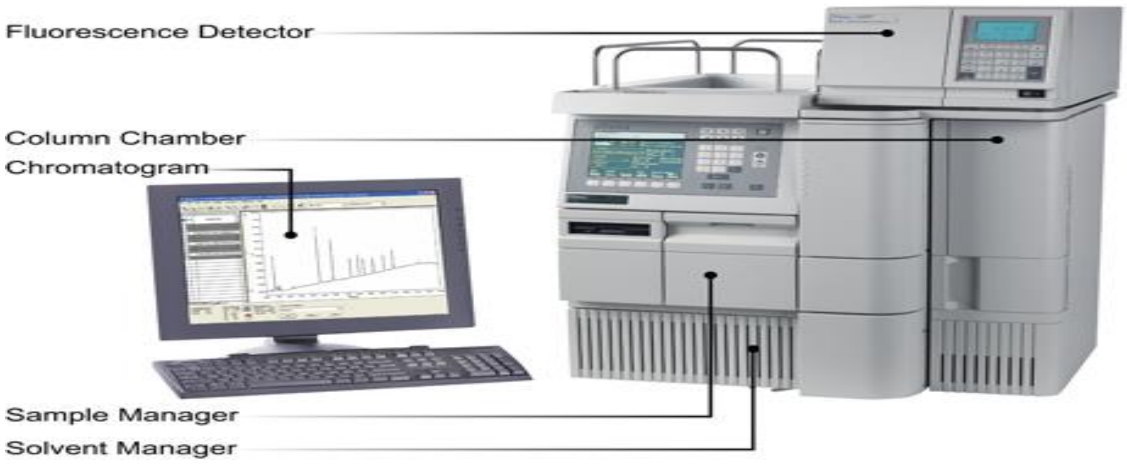

Figure 1 Typical HPLC system. Sourced from Waters (2013b)

Separation occurs due to different interaction behaviour of the analytes with the mobile liquid phase and the stationary phase within the column as the mobile phase passes through the column (Rouessac and Rouessac, 2007). Therefore, the separated compounds are introduced to the detector at different times (Figure 2).

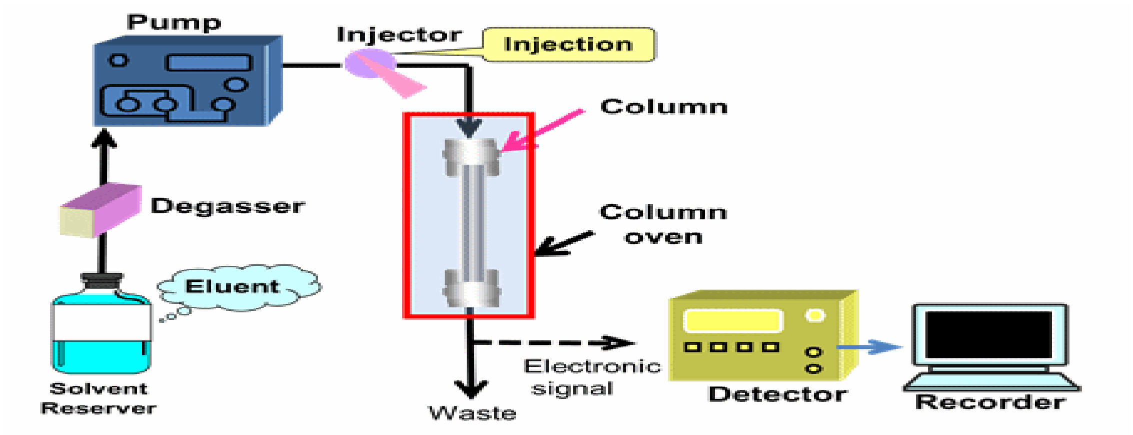

Figure 2 Components of HPLC system. Sourced from Shodex (2013)

HPLC columns can be classified into several types depending on the material used as the stationary phase e.g. reversed phase, normal phase, ion exchange, hydrophilic interaction chromatography and size exclusion column. The former is the most widely used in chemical analysis (Snyder *et al.*, 2011).

Several types of detectors may be connected to the HPLC apparatus for quantitative analysis. The most widely used detection methods are based on the optical properties of the compounds: absorbance, fluorescence and refractive index. The Ultraviolet (UV) detector, which monitors the UV absorbance of the mobile phase at the outlet of the column, is the most widely used. When an analyte passes through the detector, the absorbance intensity changes and the detector registers the presence of the analyte. A fluorescence detector provides more selectivity than UV detectors since both absorption and emission wavelengths are utilised, however, it has limited

applicability as only about 10% of organic analytes are fluorescent. The refractive index detector is designed to measure continuously the difference in the light transmission between the sample and mobile phase passing through the flow cell. It is one of the least sensitive LC detectors as it is highly affected by changes in pressure, flow rate and ambient temperature (Rouessac and Rouessac, 2007).

1.2 The use of HPLC for the analysis of azathioprine/6-mercaptopurine metabolites

Azathioprine (AZA) and 6-mercaptopurine (6-MP) are inactive prodrugs that have short half-lives in plasma, so measuring their plasma levels has no direct clinical benefit while measuring their metabolites 6-thioguanine nucleotides (6-TGN) and 6-methyl mercaptopurine (6-mMP) in red blood cells is essential in therapeutic drug monitoring (Stefan *et al.,* 2004). Moreover, intracellular accumulation of AZA/6-MP metabolites occurs over a period of 2-3 weeks which is a good indicator of long-term adherence to treatments (Rumbo, 2004).

6-TGN is considered the most active metabolite of the thiopurines (Gearry and Barclay, 2005), while 6-mMP is considered responsible for the side-effects of thiopurine therapy such as hepatotoxicity (Bruns and Stallmach, 2009; Bradford and Shih, 2011; Chouchana *et al.*, 2012). These thiopurine drugs exert their cytotoxic effect via incorporation of the active metabolite 6-TGN into DNA and RNA which result in cell apoptosis (Vikingsson *et al.,* 2009).

Genetic polymorphism and environmental factors are responsible for inter- and intra-individual variability between patients who receive the same doses of thiopurine drugs. There is an inverse relationship between thiopurine methyltransferase (TPMT) activity and TGN levels (Siegel and Sands, 2005), consequently TPMT testing prior

to treatment helps clinicians to identify patients with homozygote TPMT deficiency who may develop severe myelotoxicity due to high production of 6-TGN. In addition, other factors play a role in intraindividual variability among patients who receive the same doses of the thiopurine drug, for example caused by non-adherence to medication and diarrhoea (Erb *et al.,* 2003). Thus measuring metabolite concentrations and adjusting the AZA/6-MP dose is essential to obtain optimal therapeutic effects and decrease the risk of toxicity (Louis and Belaiche, 2003; Gearry and Barclay, 2005).

Several HPLC methods have been described for the measurement of 6-mercaptopurine and its metabolites in plasma and erythrocytes (Erdmann *et al.,* 1990; Boulieu and Dervieux, 1999), however, these studies were limited by the low recovery and complicated sample preparation procedures and require large sample volumes, e.g. 500 µL and 200 µL (Hawwa *et al.,* 2009a).

Several assays are available without sample cleaning (Boulieu *et al.,* 1993; Dervieux and Boulieu, 1998; Pike *et al.,* 2001; Erb *et al.,* 2003; Stefan *et al.,* 2004; Hawwa *et al.*, 2009a) while others have used different solid phase extraction cartridges such as SepPak® cartridges (Bonnefous *et al.,* 1992) and Hydrophilic-lipophilic-balanced (HLB) cartridges (Wang *et al.,* 2005). Such approaches enhance the sensitivity of the assays.

2 Aims and objectives

The aim of the research presented in this chapter was to develop and validate an analytical method for the quantification of AZA/6-MP metabolites in packed red blood cells and plasma using HPLC with UV detection. These compounds were 6-

thioguanine (6-TG), 6-mercaptopurine (6-MP) and 6-methyl mercaptopurine (6-mMP).

The specific objectives for this study were to:

- Develop a simple HPLC-UV microanalytical method, with adequate selectivity and sensitivity, for the quantification of the AZA/6-MP metabolites in packed RBCs and plasma obtained from children receiving azathioprine or 6-mercaptoputine.
- Validate the methodology using the current U.S. Food and Drug Administration (FDA) guidelines for bioanalytical method validation.
- Use the developed method in quantifying AZA/6-MP metabolites in children with Inflammatory Bowel Disease (IBD).

3 Materials and Methods

3.1 Reagents and chemicals

6-Thioguanine (6TG), 6-Mercaptopurine (6MP), 6-methyl Mercaptopurine (6-mMp), potassium dihydrogen phosphate and dithiotheritol (DTT) were purchased from Sigma-Aldrich (Ayrshire, UK). The chemical structures of 6-MP, 6-TG and 6-mMP are shown in Figure 3.

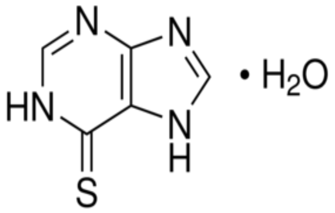

6-Mercaptopurine

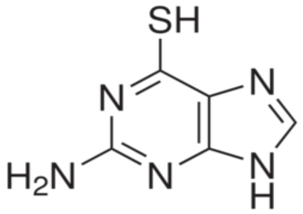

6-Thioguanine

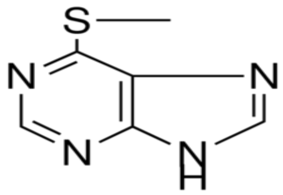

6-methyl Mercaptopurine

Figure 3 Structure of (6-MP, 6-TG, and 6-mMP)

Potassium dihydrogenphosphate (KH_2PO_4), sodium hydroxide (NaOH) were obtained from VWR international (Leicestershire, UK). HPLC grade methanol, acetonitrile and ammonia solution (NH_4OH) were obtained from VWR international (Leicestershire, UK). Formic acid was of HPLC grade, Hanks balanced salt solution (HBSS) and perchloric acid were purchased from Sigma-Aldrich (Ayrshire, United Kingdom). Oasis® MCX (Mixed-mode Cation-eXchange) solid phase extraction cartridges (1 mL/30mg) were obtained from Waters (Dublin, Ireland).

All water was HPLC grade and was prepared in house using a Millipore Direct-QTM® Water System (Millipore, Watford, UK). Filtration of HPLC mobile phases was performed using FP-Vericel™ (0.45 μm) membrane filters obtained from Sartorius (Epsom, UK).

Blank blood for method development was donated from consented healthy volunteers from the School of Pharmacy, Queen's University Belfast (QUB). School ethical approval is already in place for that purpose (School reference number: 013PMY2009).

3.2 Instrumentation and chromatographic conditions

HPLC analysis was conducted on the Waters® 2695 Separations Module connected to the Waters® 2487 Dual Wavelength Absorbance Detector. The system was controlled using Empower™ software. UV detection was used at 322nm (for the detection of 6-MP and 6-mMP) and 342 nm (for the detection of 6-TG).

Chromatographic separation was accomplished using reversed phase chromatography with gradient elution. An Atlantis® (T3) dC18 column [150mm×4.6 mm; particle size, 3 μm; Waters] protected with a guard cartridge of similar chemistry [20mm×4.6mm; particle size, 3 μm; Waters]. The mobile phase solutions were degassed and filtered prior to use.

Mobile phase A: 97% (0.02M) phosphate buffer pH 2.25, Mobile phase B: acetonitrile. The mobile phase flow was a gradient (1 mL/minute) from 97% to 80% mobile phase A in 14 minutes followed by 11 minutes re-equilibration at 97% mobile phase A. The Column temperature was 25°C and the autosampler temperature was

4°C. An aliquot of 70 µL of the extracted and cleaned sample was injected onto the HPLC.

3.3 Stock solutions and working standards

Stock solutions of 6-TG, 6-MP and 6-mMP were prepared by dissolving each compound in 2 mL of 0.1M NaOH. These stock solutions were diluted with water, to give the concentrations of 0.67 mg/mL, 1.362 mg/mL, and 1.5 mg/mL of 6-TG, 6-MP and 6-mMP respectively. All stock solutions were stored at −80°C until required. Standard solutions of the three compounds were prepared by further dilution with water (Table 1 and 2). These concentrations of the calibration standards and quality control samples were chosen as they cover the expected therapeutic RBC and plasma concentrations for each of the analytes.

Table 1 Azathioprine/6-mercaptopurine metabolites (concentration in solutions)

Analyte (µmole/L)	C1	C2	C3	C4	C5	C6	C7	C8	C9	C10
6- TG	-	0.5	0.75	1	2.5	5	10	15	17.5	20
6-MP	-	0.1	0.15	0.2	0.5	1	2	3	3.5	4
6-mMP	3.75	7.5	11.25	15	37.5	75	150	225	262.5	300

Table 2 Azathioprine/6-mercaptopurine metabolites (concentration in solutions)

Analyte (ng/mL)	C1	C2	C3	C4	C5	C6	C7	C8	C9	C10	C11
6-TG	15	30	60	90	120	300	450	600	900	1050	1200
6-MP	-	-	10	15	20	50	75	100	150	175	200
6-mMP	250	500	1000	1500	2000	5000	7500	10000	15000	17500	20000

3.4 Preparation of packed RBCs and plasma for the assay procedure

2 mL of fresh whole blank blood was centrifuged at 1000 g for 10 minutes at 4 ºC. The supernatant layer (plasma) was transferred to an Eppendorf tube (made from polypropyline) and stored at -80ºC until analysis, while the white buffy layer that is formed between the plasma and the packed red blood cell (RBCs) was removed and discarded. This layer constitutes mainly of the white blood cell (WBCs). The remaining RBCs were mixed with two volumes of HBSS for 10 seconds, centrifuged as mentioned previously, and the supernatant layer was discarded. This washing step was carried out twice. The packed RBCs were then spiked with different equimolar concentrations of AZA/6-MP metabolites to make up the packed RBCs standards and quality control samples with concentration ranges of 0.1-4 µmole/L for 6-MP, 0.5-20 µmole/L for 6-TG, and 3.75-300 µmole/L for 6-mMP. The plasma was also spiked with different equimolar concentrations of AZA/6-MP metabolites to make up plasma standards and quality control (QC) samples at concentration ranges of 10-200 ng/mL for 6-MP, 15-1200 ng/mL of 6-TG, and 250-20000 ng/mL for 6-mMP. The spiked RBCs and plasma were stored at -80ºC until further processing.

3.5 Sample preparation and extraction procedure from packed RBCs and plasma

Sample preparation of 6-TG, 6-MP and 6-mMp was based on the work of Dervieux and Boulieu (1998) and Hawwa *et al*. (2009a). In order to promote hydrolysis of thiopurine nucleotides into their corresponding bases, 100 µL of the spiked packed RBCs or 200 µL of the spiked plasma was transferred to a 1.5 mL Eppendorf tube. Then 100 microlitres of dithiotheritol (75 mg/mL) was added and the volume made up with water to 350 µL. All Eppendorf tubes were vortexed for 1 minute after which

50 microlitres of perchloric acid (700 mL/L) were added to each sample, and vortex mixed for a further 30 seconds. The tubes were then centrifuged at 13,000 × *g* for 15 minutes at 4ºC, and all the clear supernatant layer was removed and heated for 45 min on 100ºC using a heating block. After cooling, 700 µL of water was added and the solution vortex-mixed for 10 seconds before being transferred to SPE cartridges for extraction (Figure 4).

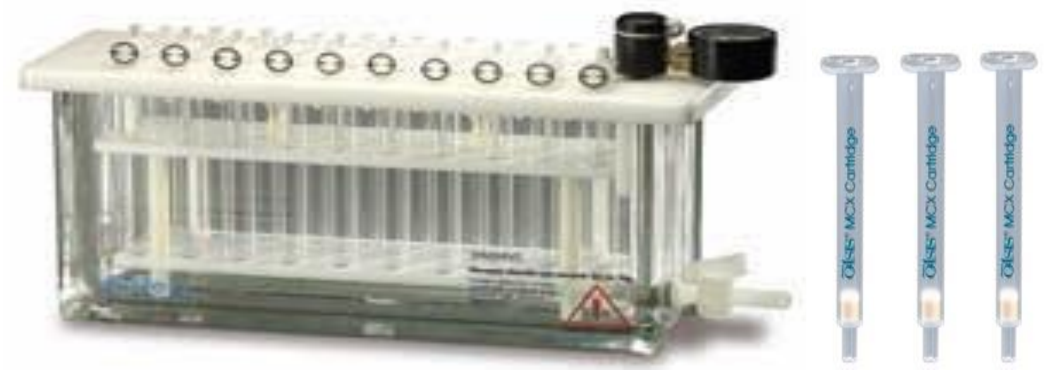

Figure 4 Solid Phase Extraction (SPE) apparatus and MCX cartridges

AZA/6-MP metabolites were extracted using Oasis® MCX cartridges (1 mL, 30 mg). According to the following procedure:

1. Condition: 1 mL methanol followed by 1 mL water.
2. Load: all the extracted sample.
3. Wash: 1 mL 2% aqueous formic acid.
4. Elute: 1 mL 5% NH_4OH in methanol.

The eluate was then evaporated under a nitrogen stream for 20 minutes at 37ºC, then reconstituted in 100 µL 0.05 M NaOH with vortex mixing for 1 minute, and transferred to an autosampler vial with insert to be injected onto the HPLC system. UV detection was at 322nm (for the detection of 6-MP and 6-mMP) and at 342 nm (for the detection of 6-TG).

3.6 Method validation

The parameters that have been assessed for the purpose of method validation were as follows: selectivity, linearity, limit of detection (LOD) and lower limit of quantification (LLOQ), accuracy and precision, recovery and stability.

(a) Selectivity

Selectivity is the ability of the analytical method to distinguish and measure an analyte in the presence of other compounds (interferences). This was conducted using five different sources of blood from five volunteer subjects.

Selectivity of the HPLC analytical method was demonstrated by analysing blank packed RBCs or plasma samples versus packed RBCs or plasma samples that were spiked with AZA/6-MP metabolites (n=5) to a final concentration that equalled the determined lower limit of quantification.

The effect of concomitant medication was assessed also by injecting standard solutions of high concentrations of some drugs which may be administered with AZA/6-MP in patients with IBD and the presence of any interferences at the retention time of the target analytes were checked.

(b) Linearity

The linearity of the developed method was assessed by creating calibration curves for each 6-MP, 6-TG, and 6-mMP individually over 5 consecutive days. Calibration plots were constructed for peak area versus the analyte concentrations to evaluate the relationship between the two parameters. Linear regression analysis was used to find out the intercept, slope and correlation coefficient of the calibration curves.

(c) Limit of detection and limit of quantification

The LOD is the lowest concentration that can be observed (i.e. forms a diversifiable peak) from the measurement of a sample that contains the analyte. The lower limit of quantification (LLOQ) is the lowest quantity of the analyte that can be determined (quantified) with acceptable precision and accuracy under the determined operational conditions.

Usually LOD is the concentration at which the signal equals 3 times the baseline signal, and LLOQ is the concentration at which the signal equals 10 times the baseline signal. In this study, the LOD and LLOQ were calculated according to the recommendations of the International Conference on Harmonisation (ICH) using the following equations (ICH, 1996):

$LOD=3.3\sigma/S$

$LLOQ=10\sigma/S$

Where σ is the standard deviation of the response, and S is the slope of the calibration curve.

(d) Accuracy and precision

The degree of proximity of the determined value to the true value (concentration) of the analyte is known as the accuracy of the analytical method. Precision of an analytical method is defined as the closeness of repeated individual measures of the analyte (FDA, 2013). Inter- and intra-day accuracy and precision of the developed method were calculated using the data collected from the within day and between days calibration curves. Four concentrations were tested in terms of accuracy and precision: LLOQ, lower, middle and high quality control (QC) concentrations.

Intra-day (in single day) accuracy and precision were calculated using five replicates at each concentration from a single day run, while inter-day accuracy and precision were calculated using one replicate at each concentration over five consecutive days.

The accuracy was expressed as the mean percent relative error (RE%), while precision was expressed as the percent coefficient of variation (CV%). An accepted accuracy or precision according to the U.S. Food and Drug Administration (FDA) guidelines should be within 15% of the actual value for the quality control samples. However, for the lower limit of quantification, an accepted deviation should be equal or less than 20% (FDA, 2013).

(e) Recovery

The recovery of an analyte was calculated as the ratio of the detector response for a certain concentration of 6-TG, 6-MP and 6-mMP in packed red blood cells or plasma to the detector response obtained for the aqueous solution containing the same concentration of the compounds. Recovery is not required to be 100%, however, it should be consistent and reproducible. Recovery was tested at the three QC concentrations (n= 6 at each concentration).

(f) Stability

The stability of AZA/6-MP metabolites over a 3 month period was conducted to make sure that consistent results were produced from stored samples. Samples were tested at the high, mid and low QC concentrations (n=5 at each concentration).

3.3.7 Statistical methods and data analysis

All HPLC data were collected and analysed on Empower® software (Waters Corporation, USA). Linearity was calculated using linear regression analysis. Means,

standard deviations and CV% were calculated using Microsoft® Excel 2007 (Microsoft Corporation, USA).

4 Results and discussion

4.1 Sample processing and chromatographic conditions

Initial analytical method development in the present study was based on previous published methods (Dervieux and Boulieu, 1998; Hawwa *et al*., 2009a) for simultaneous determination of AZA/6-MP metabolites in packed RBCs.

Chromatographic conditions, including column temperature of 25ºC and phosphate buffer composition (20mM, pH 2.25) employed by Hawwa *et al.* (2009a) were incorporated into the analytical method. Different phosphate buffers prepared at pH 2.25, 2.8, 3.2 were also tested during method optimisation, however, phosphate buffer at pH 2.25 was found to be the most suitable in obtaining good resolution for all peaks of interest as suggested by Hawwa *et al.* (2009a).

Initially, a mobile phase consisting of a mixture of methanol (1%), acetonitrile (3%) and dihydrogen phosphate buffer (96%) delivered isocratically at a flow rate of 1 mL/min, as suggested by Hawwa *et al* (2009a) was used. This enabled acceptable separation of all compounds. To achieve better resolution of the compounds, however, a gradient elution approach was applied.

As is the case with the majority of the assay methods employed for the quantification of AZA/6-MP metabolites, measured levels of 6-TGN were based on the conversion of the total RBC 6-TGN (mono-, di- and tri-phosphate) nucleotides into the free 6-TG base, during acid hydrolysis. Each of these nucleotides forms were not quantified separately. Likewise, measurement of 6-mMP in RBCs represented total 6-mMP(R) rather than each form of nucleotides. On the other hand, plasma levels represented the free bases that circulate in plasma (Hawwa *et al.*, 2009a).

The determination of 6-mMP without the hydrolysis step is technically very difficult because this compound is not stable at the low pH value which is needed for the hydrolysis of thiopurine nucleotides into their free bases (Dervieux and Boulieu, 1998). During the hydrolysis step, 6-mMP was converted completely into 4-amino-5-(methylthio) carbonyl imidazole (AMTCI) which is easily measured (quantified) under the same chromatographic conditions used for other bases (Dervieux and Boulieu, 1998; Hawwa *et al.*, 2009a).

The use of DTT as a thiol group protector aid increases the recovery, and especially in the low pH media needed for hydrolysis (Dervieux and Boulieu, 1998; Hawwa *et al.*, 2009a), thus DTT was added one minute before addition of perchloric acid.

In order to obtain the best recovery for AZA/6-MP metabolites, all available Waters Oasis® cartridges (HLB: Hydrophilic-Lipophilic Balance Sorbent reversed-phase sorbent, MCX: Mixed-mode Cation-eXchange and reversed-phase sorbent, MAX: Mixed-mode Anion-eXchange and reversed-phase sorbent, WCX: Mixed-mode Weak Cation-eXchange and reversed-phase sorbent, WCX: Mixed-mode Weak Cation-eXchange and reversed-phase sorbent) were tested using the Oasis 2x4 method developed by Waters.

Best recovery was obtained using the MCX cartridges. Further optimisation was carried out using the MCX SPE cartridges in order to get a clean matrix and highest recovery. At the beginning, the procedure was as follows:

1. Condition: 1 mL methanol followed by 1 mL water.
2. Load: all the supernatant resulting after protein precipitation and hydrolysis.
3. Wash1: 1 mL 5% aqueous formic acid.
4. $Wash_2$: 1 mL absolute methanol.

5. Elution: the elution step was 1 mL of one of the following: 0, 10, 20, 30, 40, 50, 60, 70, 80, 90 or 100% (water: methanol) with 5% NH_4OH.

It was found that recovery increased when the methanol washing step was omitted from the procedure, and when using 30% methanol in water as the elution step, however, 100% methanol was finally used for elution step to simplify the preparation procedure as the later needs 20 minutes drying while the former required 45 minutes.

Other attempts to increase recovery, i.e. using NaOH (0.1M) for the elution step, were tested as it is used for stock preparation. A low concentration of NaOH (0.05 M) was eventually chosen because the compounds of interest are soluble in dilute basic solution with slower decomposition. The chromatograms for the AZA/6-MP metabolites, extracted from packed RBCs and plasma, together with their retention times, are shown in Figures 5and 6.

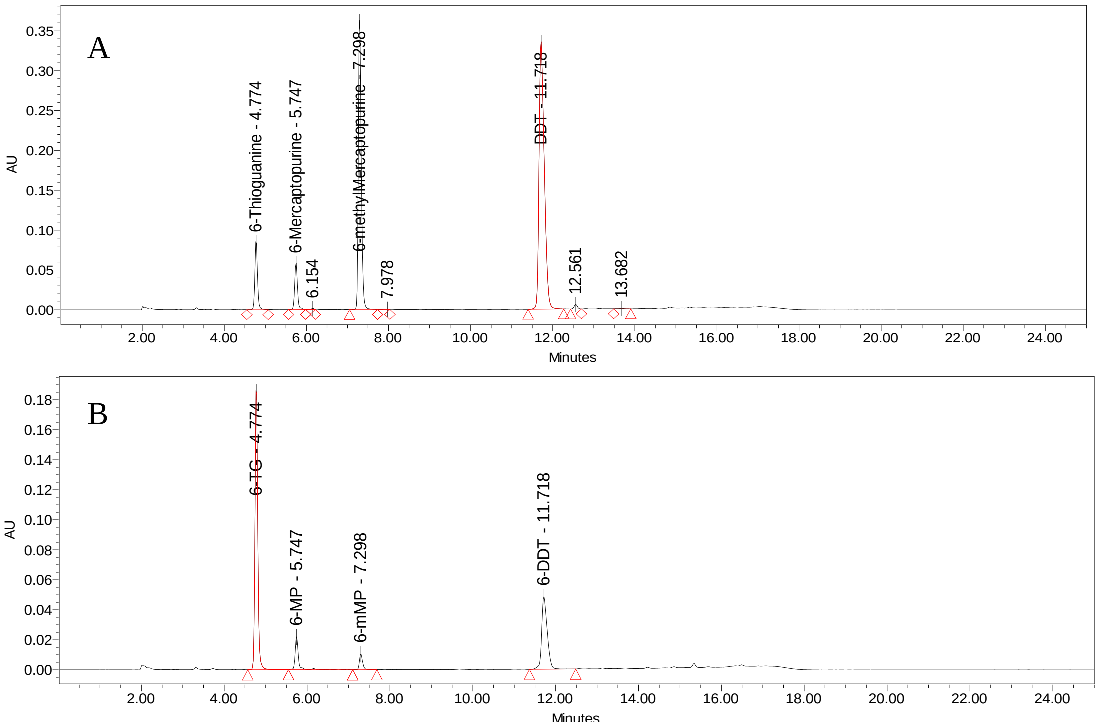

Figure 5 Chromatogram of packed RBCs spiked with 6-TG, 6-MP, and 6-mMP at the level of 20, 4, 300 µM, respectively and detected at A) 322nm and B) 342nm

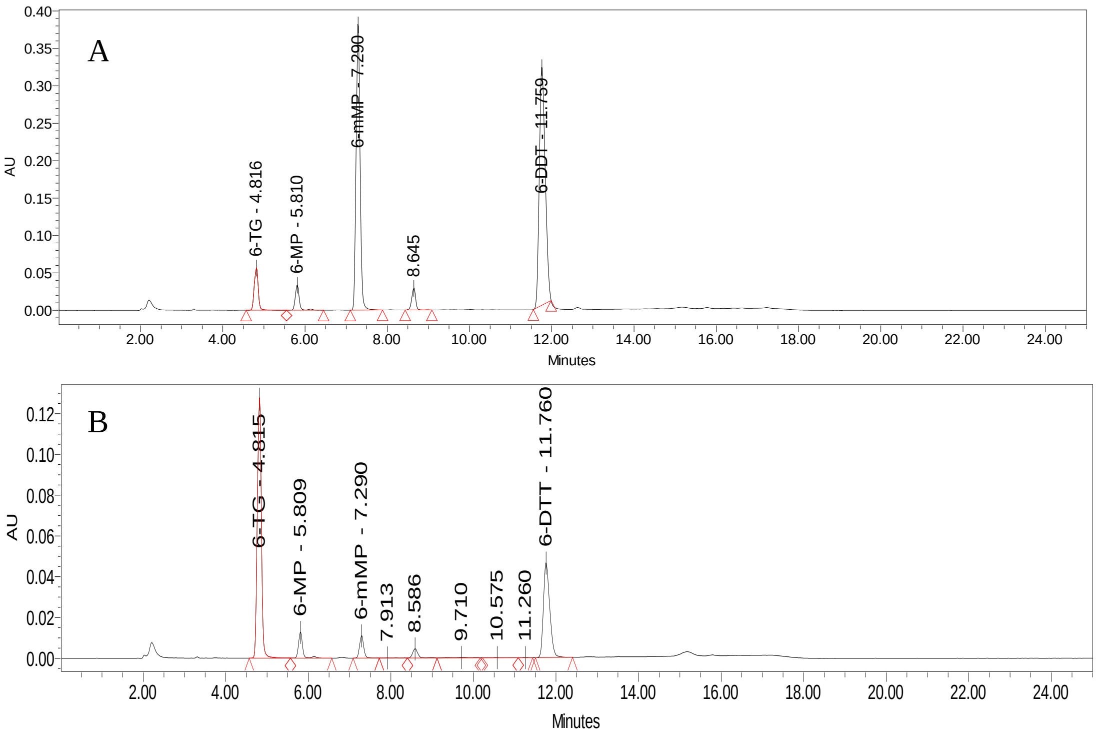

Figure 6 Chromatogram of plasma spiked with 6-TG, 6-MP, and 6-mMP at the level of 1200, 200, 20000 ng/mL, respectively and detected at A) 322nm and B) 342nm

4.2 Results of method validation

Subsequent to the development and optimisation of the extraction and chromatographic conditions, validation of the assay was performed.

(a) Selectivity

Good peak resolution was achieved for AZA/6-MP metabolites utilising the chromatographic conditions detailed above. No interfering peaks were detected in the extracted blank packed RBCs or plasma chromatograms when compared with chromatograms of extracted packed RBCs or plasma samples spiked with AZA/6-MP metabolites at LLOQ as shown in Figures 7 and 8. This indicated that the method presented good selectivity and the AZA/6-MP metabolites were not affected by the presence of interferences or endogenous compounds. In addition, selectivity of the analytical method was also deemed acceptable using blank packed RBCs (Figure 9) and blank plasma samples from five different blood sources (Figure 10).

After referring to patient's hospital medical notes, it was found that most of the patients included in the study presented in Chapter 5 were taking, in addition to AZA/6-MP, the following drugs: metronidazole, ranitidine and omeprazole. An injection of standard solutions of each drug was conducted and this revealed that they were eluted at different times from the AZA/6-MP metabolites, thus no interferences were found to affect the quantification of the target analytes (chromatograms are shown in Figure 11).

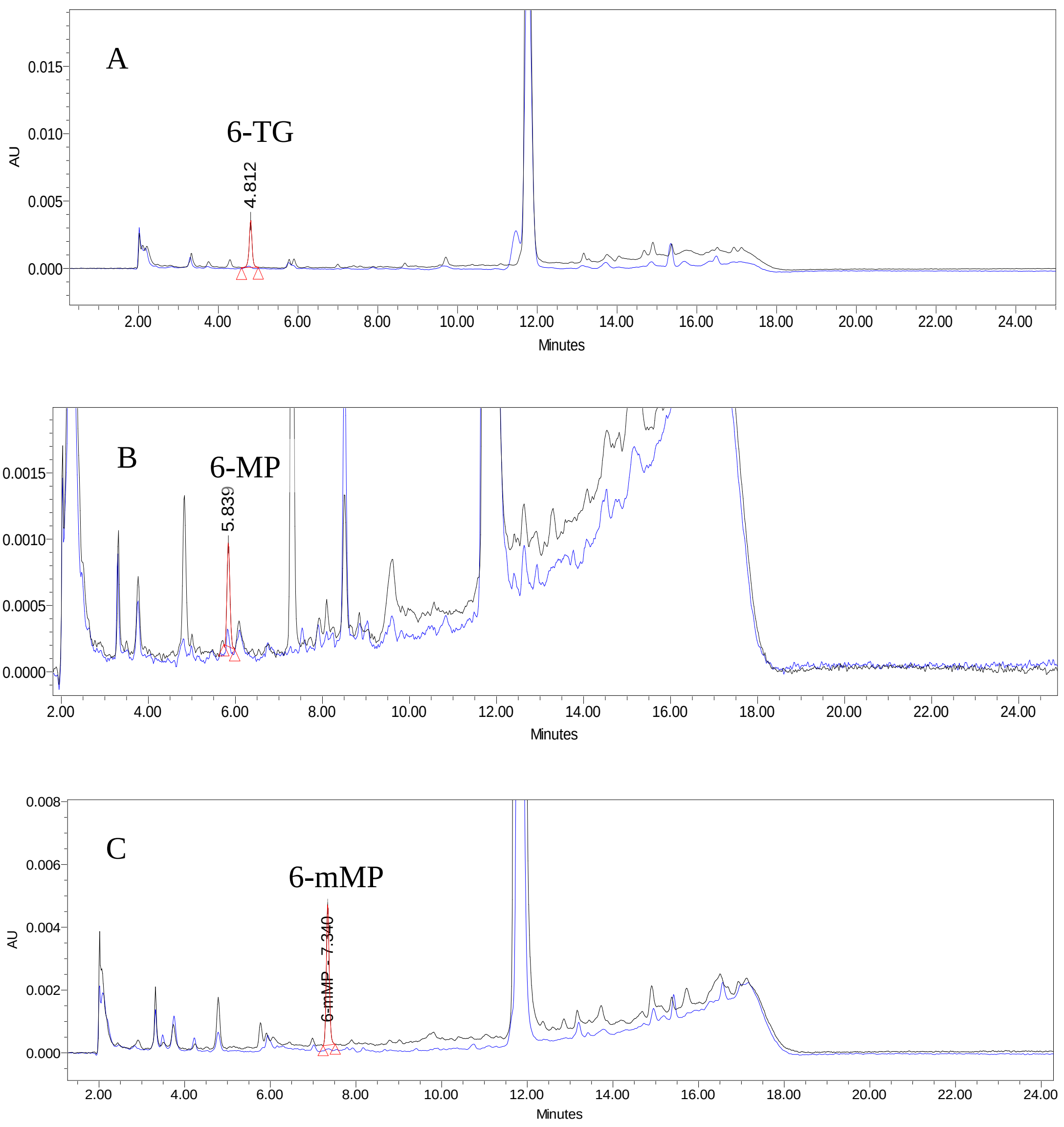

Figure 7 Overlay chromatograms of a blank packed red blood sample (blue) and a packed red blood sample spiked with 6-TG (A), 6-MP (B), and 6-mMP (C) at the LLOQ level of 0.5, 0.1, 3.75 µM, respectively and detected at 322nm for 6-MP and 6-mMP and at 342nm for 6-TG

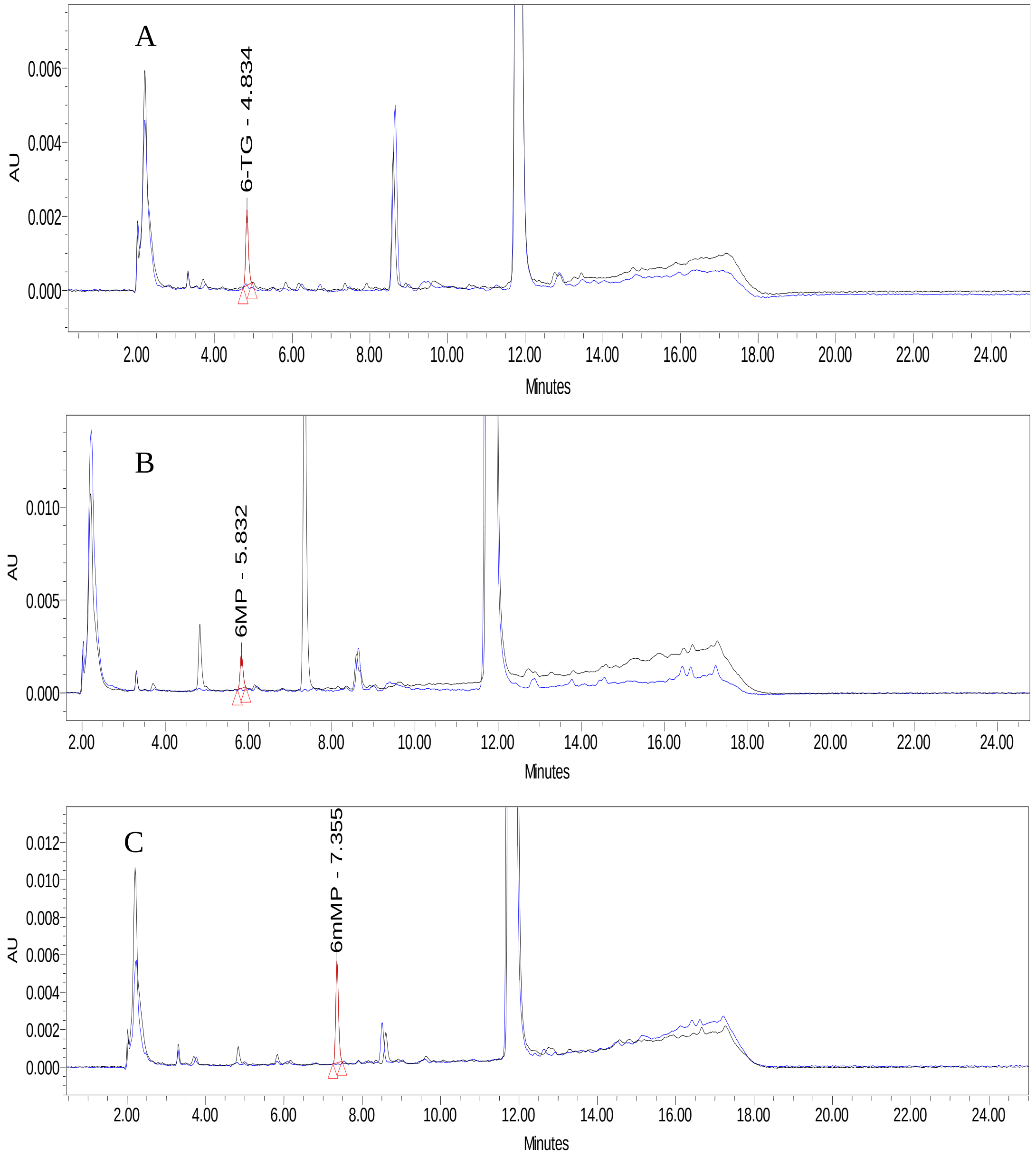

Figure 8 Overlay chromatograms of a blank plasma sample (blue) and a plasma sample spiked with 6-TG (A), 6-MP (B), and 6-mMP (C) at the LLOQ level of 15, 10, 250 ng/mL, respectively and detected at 322nm for 6-MP and 6-mMP and at 342nm for 6-TG

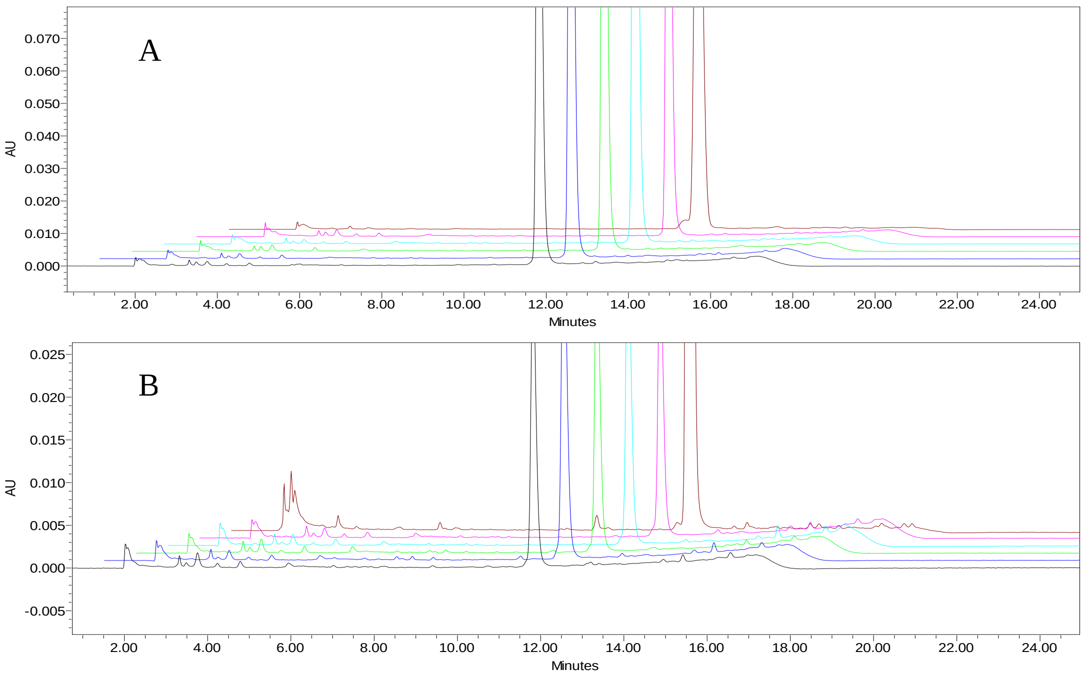

Figure 9 Overlay chromatograms of analysed blank packed red blood from 5 different sources detected at 322nm (a) and at 342nm (b)

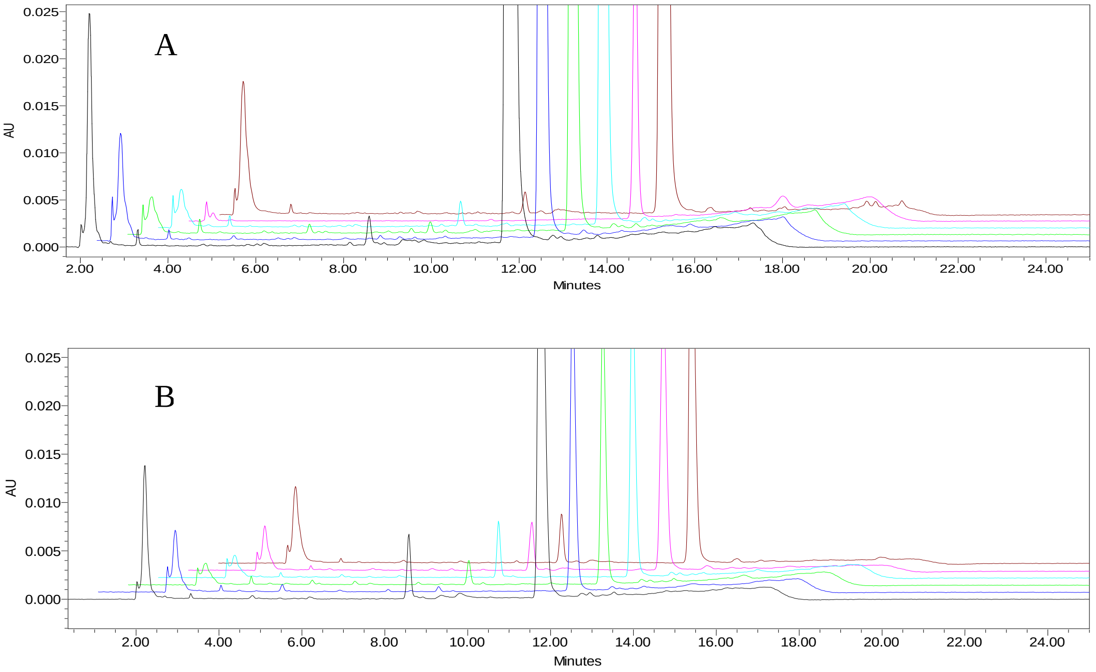

Figure 10 Overlay chromatograms of analysed blank plasma from 5 different sources detected at 322nm (A) and at 342nm (B)

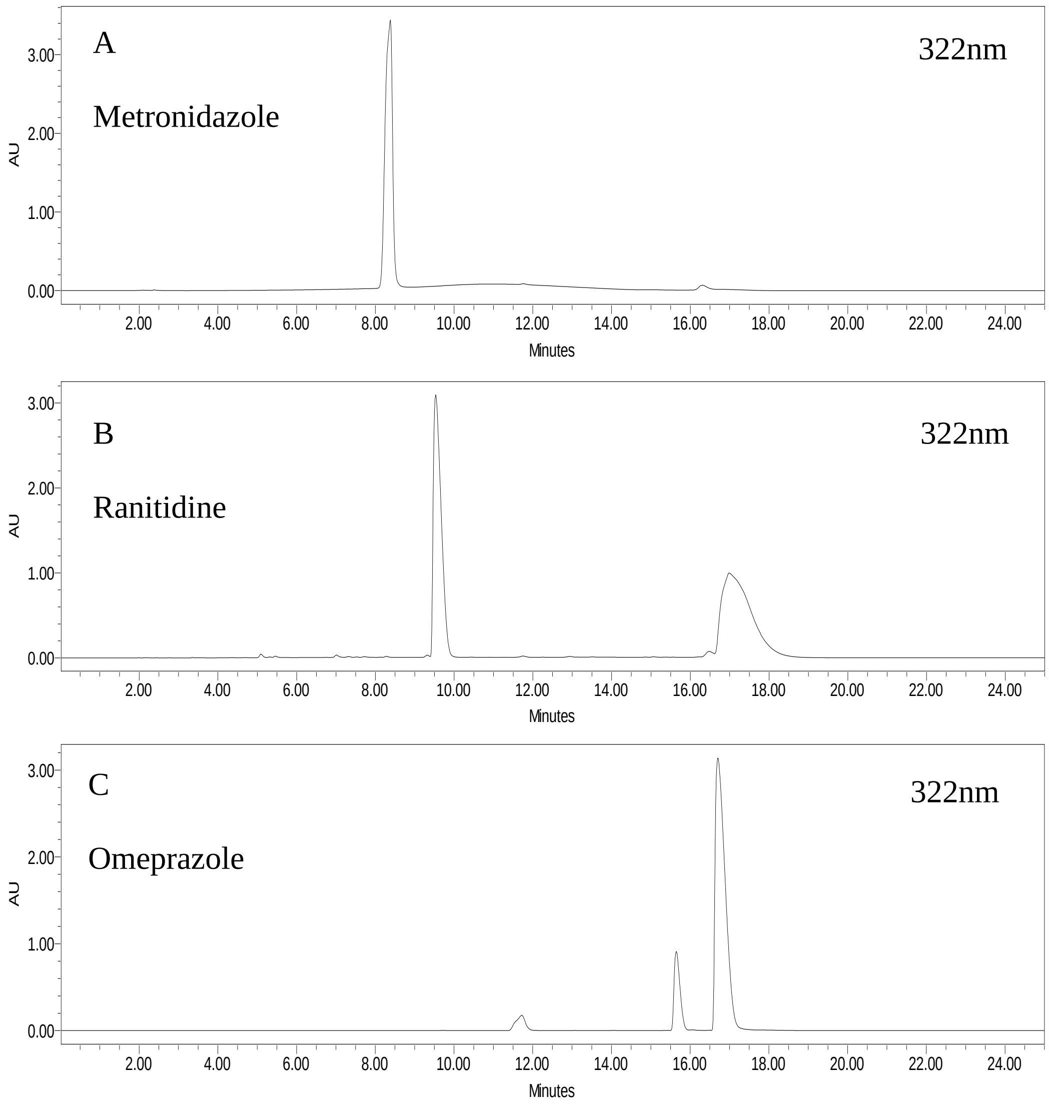

Figure 11 Chromatograms of solutions containing A) metronidazole, B) ranitidine and C) omeprazole. All solutions were processed according the method developed for the quantification of AZA/6-MP metabolites

(b) Calibration curve and linearity

A linear regression model was used to describe the concentration-response relationship. According to that model, the back calculated values from the QC responses were the most accurate and reliable. The deviation from the best fitted regression line was less than 15% for all the calibration point except the deviation for LLOQ which was less than 20%.

The linearity of the response was found to be acceptable and reproducible over the calibration curve range.

(c) Limits of detection and quantification

The LOD and LLOQ were calculated using the equations described previously and were found to be as follows for AZA/6-MP metabolites in packed RBCs (Table 3) and in plasma (Table 4).

Table 3 Limit of detection (LOD) and lower limit of quantification (LLOQ) for AZA/6-MP metabolites in packed red blood cells

Calibration curve ID	6-TG		6-MP		6-mMP	
	LOD (µM)	LLOQ (µM)	LOD (µM)	LLOQ (µM)	LOD (µM)	LLOQ (µM)
**1**	0.141	0.464	0.031	0.102	1.741	5.745
**2**	0.213	0.704	0.030	0.098	1.240	4.093
**3**	0.166	0.547	0.032	0.104	1.545	5.099
**4**	0.184	0.606	0.033	0.109	1.204	3.972
**5**	0.184	0.606	0.039	0.131	1.087	3.59
**Mean**	**0.178**	**0.585**	**0.033**	**0.109**	**1.363**	**4.499**
**SD**	**0.027**	**0.088**	**0.004**	**0.013**	**0.270**	**0.892**
**CV%**	**15.169**	**15.042**	**12.120**	**11.930**	**19.810**	**19.830**

SD: Standard deviation. CV%: Coefficient of variation = (SD×100)/mean

Table 4 Limit of detection (LOD) and lower limit of quantification (LLOQ) for AZA/6-MP metabolites in plasma

Calibration curve ID	6-TG		6-MP		6-mMP	
	LOD (ng/mL)	LLOQ (ng/mL)	LOD (ng/mL)	LOQ (ng/mL)	LOD (ng/mL)	LLOQ (ng/mL)

**1**	4.761	15.710	1.845	6.09	56.73	187.20
**2**	4.009	13.230	2.133	7.04	55.15	182.00
**3**	3.391	11.190	2.091	6.9	58.20	192.07
**4**	3.021	9.970	2.479	8.18	70.24	231.8
**5**	3.221	10.630	2.567	8.47	65.39	215.8
**Mean**	**3.680**	**12.146**	**2.223**	**7.336**	**61.142**	**201.774**
**SD**	**0.708**	**2.336**	**0.296**	**0.978**	**6.419**	**21.189**
**CV%**	**19.24**	**19.23**	**13.315**	**13.332**	**10.500**	**10.501**

SD: Standard deviation. CV%: Coefficient of variation = (SD×100)/mean

The validated LLOQ values using the developed method for packed RBCs were 0.5 µM, 3.75 µM and 0.1 µM and in plasma were 15 ng/mL, 250 ng/mL and 10 ng/mL for 6-TG, 6-mMP and 6-MP respectively. The LLOQ values in packed red blood cells corresponds to quantification limit of 36 pmol/ 8 × 10^8 erythrocytes for 6-TG, 7 pmol/ 8 × 10^8 erythrocytes for 6-MP and 275 pmol/ 8 × 10^8 erythrocytes for 6-mMP, assuming that 1.1 × 10^9 erythrocytes per 100 µL of packed erythrocytes (Dervieux *et al.*, 2005). Inter- and intra-day precision and accuracy values at this concentration were adequate with CV% and RE% values falling within the allowed range (≤20%).

(d) Accuracy and precision

Inter- and intra-day accuracy and precision (LLOQ, low QC, middle QC and high QC) data for packed RBCs and plasma are summarised in Table 5 and 6 respectively.

All inter- and intra-day accuracy and precision measures obtained from the 5-day validation results were satisfactory according to the FDA guideline (CV% and RE%

values within 15% of the actual value except for LLOQ which should not deviate by more than 20%).

Table 5 Precision and accuracy of quantification of AZA/6-MP metabolites in packed RBCs (n=5)

Compound	Nominal concentration (µM)	Intra-day (n=5)			Inter-day (n=5)		
		Accuracy		Precision	Accuracy		Precision
		Mean±SD*	**RE%	***CV%	Mean±SD*	**RE%	***CV%
**6-TG**	LLOQ (O.5)	0.543±0.030	8.60	5.52	0.515±0.043	3.00	8.35
	Low QC (1)	0.930±0.109	-7.00	11.72	1.010±0.057	1.00	5.64
	Middle QC (5)	5.237±0.191	4.74	3.65	4.920±0.394	-1.60	8.01
	High QC (15)	14.705±0.300	-1.97	2.04	14.641±1.194	-2.39	8.16
**6-MP**	LLOQ (O.1)	0.096±0.013	-4.00	13.54	0.103±0.013	3.00	12.62
	Low QC (0.2)	0.196±0.013	-2.00	6.63	0.195±0.013	-2.50	6.67
	Middle QC (1)	1.115±0.076	11.5	6.82	1.007±0.092	0.720	9.14
	High QC (3)	3.327±0.094	10.90	2.83	2.983±0.372	-0.567	12.47
**6-Mmp**	LLOQ (3.75)	4.490±0.571	19.73	12.69	3.428±0.453	-8.59	13.22
	Low QC (15)	12.768±0.652	-14.88	5.11	15.296±2.19	1.97	14.32
	Middle QC (75)	80.864±4.37	7.82	5.40	74.898±4.30	-0.14	5.74
	High QC (225)	225.214±20.13	0.10	8.94	227.324±16.52	1.03	7.26

*SD: Standard deviation. **RE%: Relative error = (nominal concentration-measured concentration) × 100/nominal concentration
***CV%: Coefficient of variation = (SD×100)/mean

Table 6 Precision and accuracy of quantification of AZA/6-MP metabolites in plasma (n=5

Compound	Nominal concentration (ng/mL)	Intra-day (n=5)			Inter-day (n=5)		
		Accuracy		Precision	Accuracy		Precision
		Mean±SD*	**RE%	***CV%	Mean±SD*	**RE%	***CV%
**6-TG**	LLOQ (15)	17.22±3.40	14.80	19.74	14.90±1.90	-0.667	12.75
	Low QC (60)	66.80±6.94	11.33	10.39	64.65±2.06	7.75	3.19
	Middle QC (300)	254.93±19.90	-15.0	7.80	300.95±27.71	0.317	9.20
	High QC (1050)	1011.21±61.62	-3.69	6.09	1064.70±32.95	1.40	3.09
**6-MP**	LLOQ (10)	11.09±1.57	10.90	14.16	11.32±0.696	13.20	6.15
	Low QC (50)	42.5±4.27	-15.0	10.05	48.88±4.45	-2.24	9.10
	Middle QC (100)	95.73±2.44	-4.27	2.55	98.36±0.907	-1.64	0.92
	High QC (175)	168.12±5.19	-3.93	3.08	168.32±9.59	-3.82	5.69
**6-mMP**	LLOQ (250)	236.38±28.97	-5.45	12.26	267.30±20.55	6.92	7.69
	Low QC (1000)	1067.16±131.55	6.72	12.33	1072.73±50.05	7.273	4.67
	Middle QC (5000)	4261.17±564.95	-14.78	13.26	5089.58±246.73	1.792	4.85
	High QC (17500)	17567.48±490.57	0.39	2.79	17158.38±318.63	-1.952	1.86

*SD: Standard deviation. **RE%: Relative error = (nominal concentration-measured concentration) ×100/nominal concentration
***CV%: Coefficient of variation = (SD×100)/mean

(e) Recovery

The recovery of the method was satisfactory to ensure that AZA/6-MP metabolites can be accurately analysed at the three QCs levels. The recovery of the each analyte was reproducible, reliable and consistent. Tables 7 and 8 show the recovery of AZA/6-MP metabolites at the three QC levels from packed red blood cells and plasma.

Loss of the compounds during the cleaning procedure may explain the lower recovery values of the three compounds when compared with those reported by Dervieux and Boulieu (1998), Oliveira *et al.* (2004) and Hawwa *et al.* (2009a), in which samples were injected directly after extraction, i.e. without cleaning.

Table 7 Recovery of AZA/6-MP metabolites from packed red blood cells

Compound	Concentration (µM)	%Recovery (n=6)		% Recovery (for the 3 QCs)	
		Mean ± SD	CV%	Mean ± SD	CV%
**6-TG**	**1**	46.27±3.26	7.05	46.73±2.99	6.40
	**5**	44.00±2.26	5.14		
	**15**	49.92±1.71	3.43		
**6-MP**	**0.2**	66.52±7.10	10.67	65.06±1.73	2.66
	**1**	63.15±3,51	5.56		
	**3**	65.49±1.85	2.82		
**6-mMP**	**15**	64.57±2.56	3.96	64.99±4.36	6.71
	**75**	60.86±4.46	7.33		
	**225**	69.54±5.22	7.50		

SD: Standard deviation. CV%: Coefficient of variation = (SD×100)/mean

Table 8 Recovery of AZA/6-MP metabolites from plasma

Compound	Concentration (ng/mL)	%Recovery (n=6)		% Recovery (for the 3 QCs)	
		Mean ± SD	CV%	Mean ± SD	CV%
**6-TG**	**60**	63.04±1.16	1.84	66.15±2.72	4.11
	**300**	68.10±1.00	1.47		
	**1050**	67.31±1.41	2.09		
**6-MP**	**50**	64.73±2.78	4.29	64.75±0.34	0.53
	**100**	65.10±4.00	6.14		
	**175**	64.42±3.62	5.62		
**6-mMP**	**1000**	66.52±2.92	4.39	65.09±1.74	2.67
	**5000**	63.15±2.60	4.18		
	**17500**	65.62±4.12	6.28		

SD: Standard deviation. CV%: Coefficient of variation = (SD×100)/mean

(f) Stability

The recovery of three replicates of low, middle and high QC concentrations of AZA/6-MP metabolites were assessed 6 times over a 3 month period, and during which they were stored in -80ºC. All compounds were stable over 3 months (Figures 12 and 13).

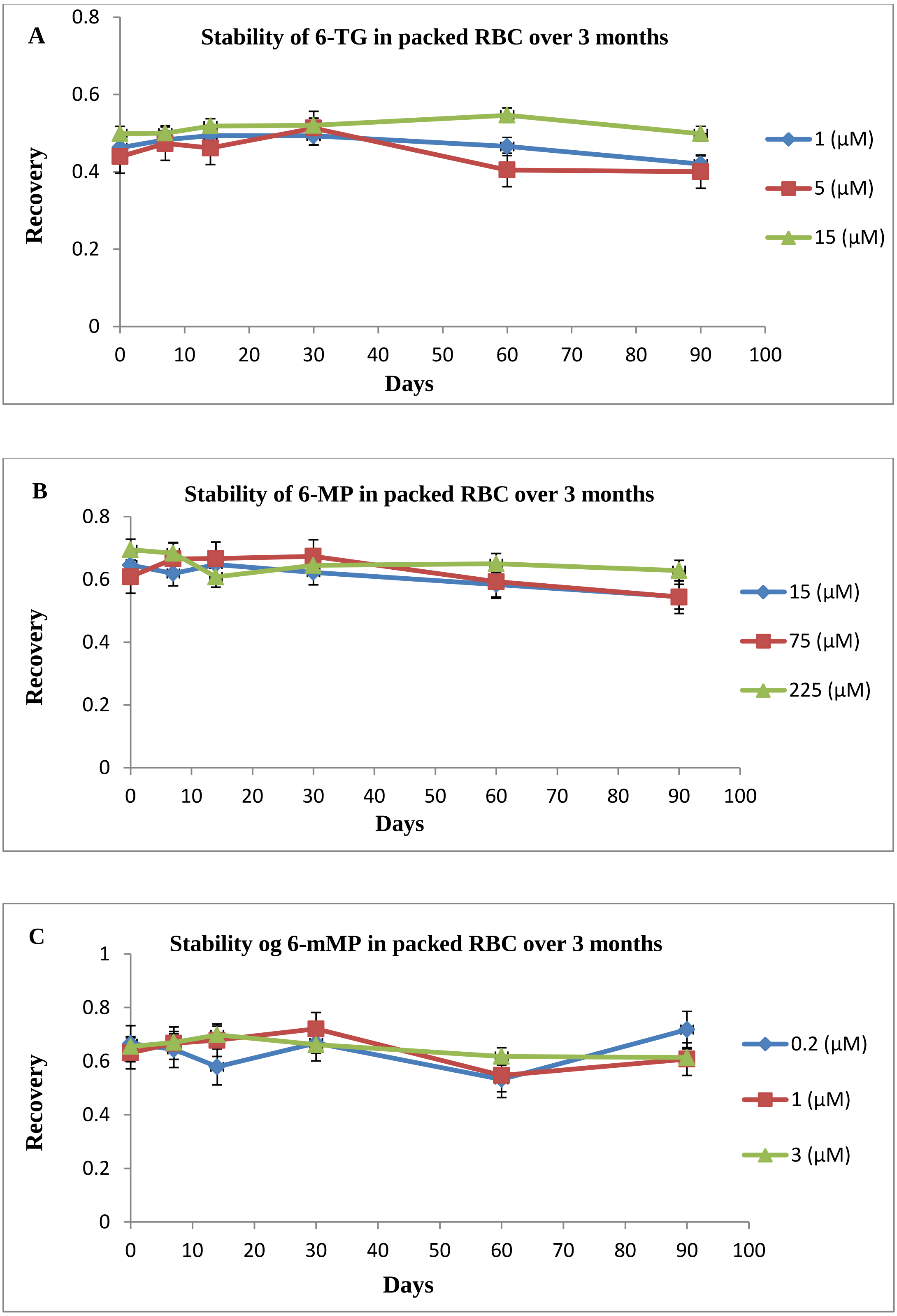

Figure 12 Stability of AZA/6-MP metabolites in RBCs over 3 months, A) 6-TG, B) 6-MP and C) 6-mMP

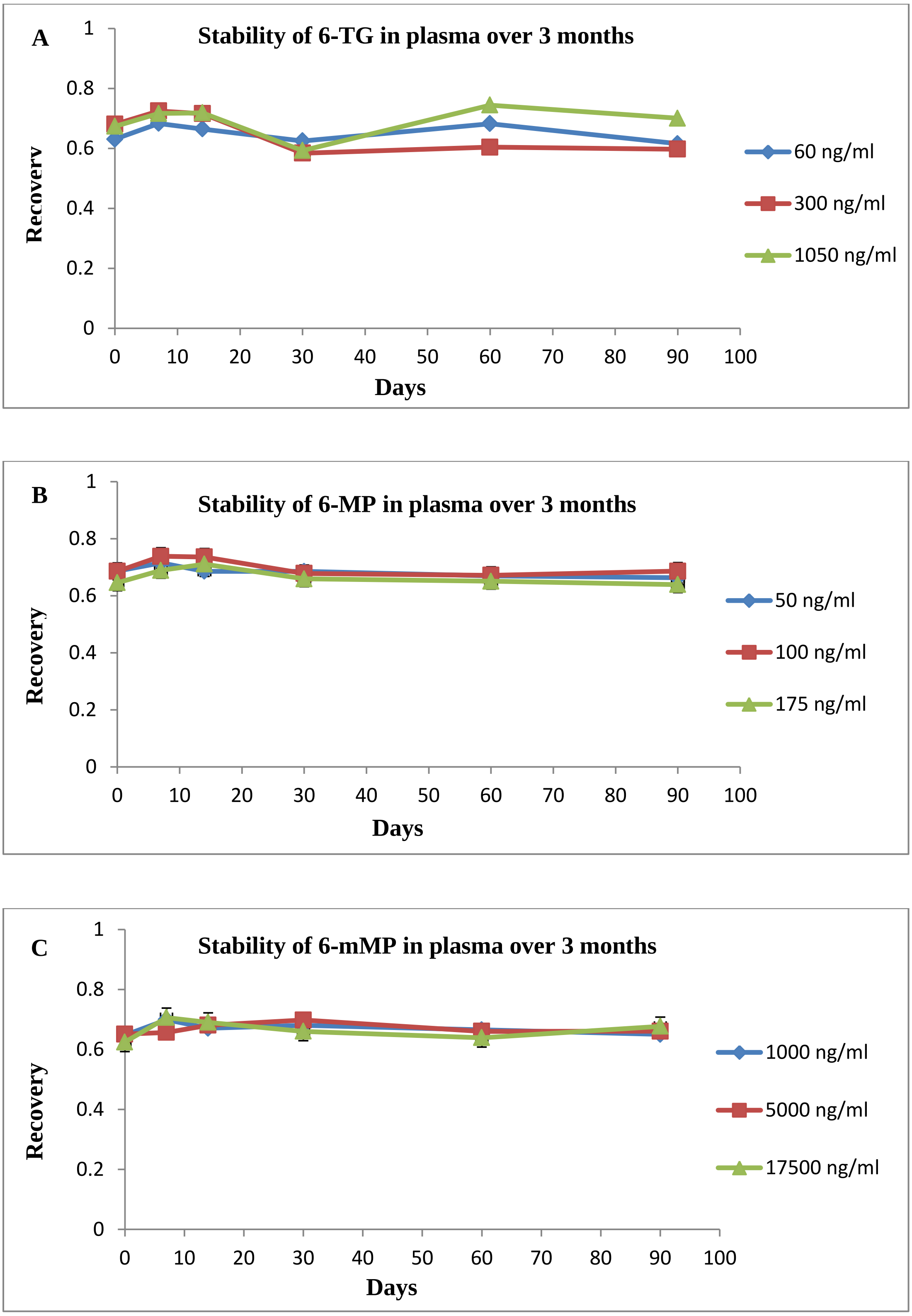

Figure 13 Stability of AZA/6-MP metabolites in plasma over 3 months, A) 6-TG, B) 6-MP and C) 6-mMP

4.3 Application of the developed method

The developed method was applied to the analysis of azathioprine/6-mercaptopurine metabolites in packed RBCs and plasma samples collected from children with inflammatory bowel disease. All patients' samples were analyzed within two weeks of blood collection.

Each analytical run included QC samples, calibration standards and a batch of processed patient samples. Calibration curves were generated for 6-MP, 6-TG and 6-mMP in each analytical run.

Chromatograms of RBC and plasma samples from two IBD patients are shown in Figures 14 and 15 as examples. The first patient was a 13 year old male patient who received 125 mg of azathioprine once daily. The RBC sample from the first patient was withdrawn 3.5 hrs after the dose. The concentrations of 6-TG and 6-mMP found in the RBC sample were 6.01 and 45.58 µM respectively.

The second patient was a 14 year old female patient, who received 50 mg of 6-MP. Her plasma sample was taken 3 hrs after taking the dose. The presence of 6- MP was clear in the plasma sample and its concentration was 63.09 ng/mL.

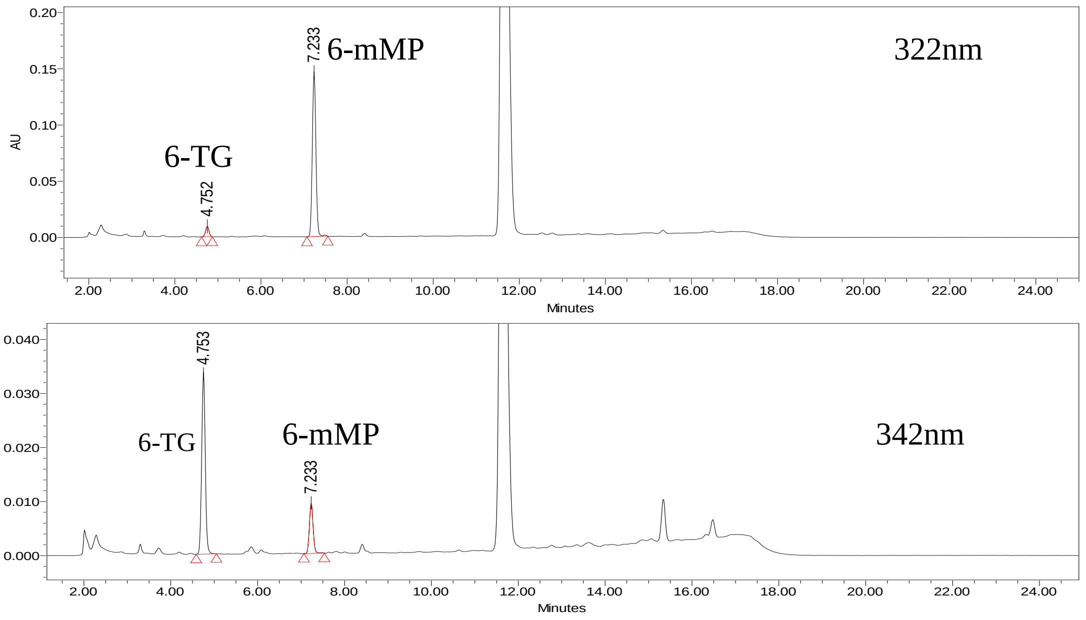

Figure 14 Chromatograms of azathioprine metabolites in RBC sample obtained from a male patient (age 13 years) who received 125 mg of azathioprine daily. The sample was taken 3.5 hours after taking his dose

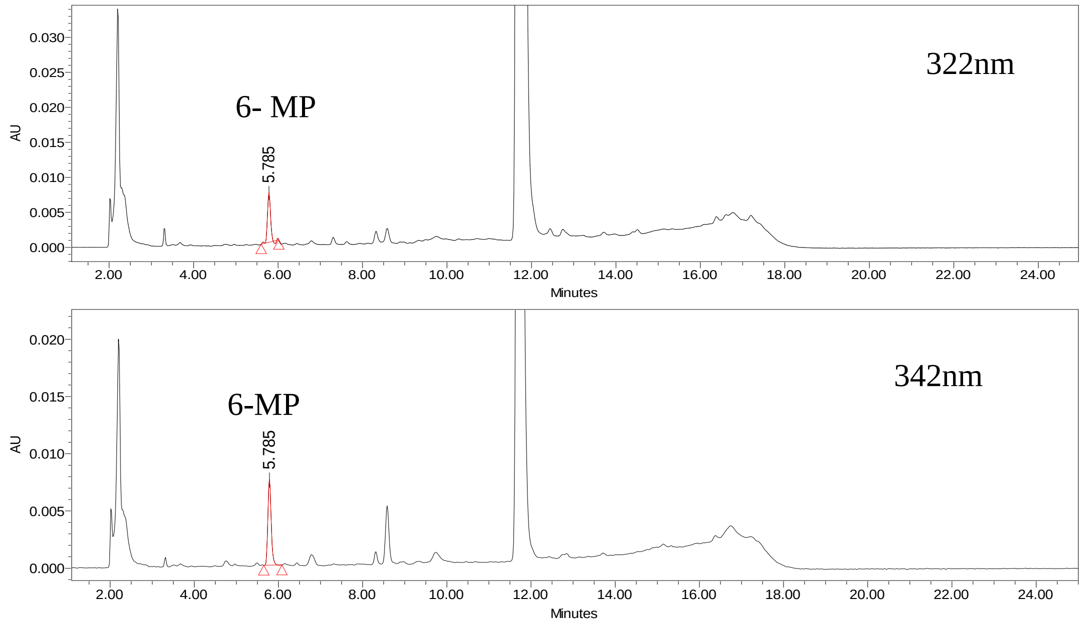

Figure 15 Chromatograms of mercaptopurine in plasma sample obtained from a female patient (age 14 years) who received 50 mg of mercaptopurine daily. The sample was taken 3 hours after taking his dose

5 Conclusions

The following conclusions can be drawn from the present study:

- A simple analytical method for simultaneous analysis of azathioprine/6-mercaptopurine metabolites in packed RBCs and plasma has been developed, optimised and validated successfully according to the recommended guidelines using low volume biological samples (100 µL of packed red blood cells or 200 µL of plasma).
- During method development, achieving a clean sample with best recovery for 6-MP, 6-TG and 6-mMP proved to be challenging. However, the three compounds could be quantified using the analytical method developed with satisfactory accuracy and precision.
- The validated microanalytical method has been successfully applied in analysing packed RBCs obtained from paediatric patients with inflammatory bowel disease.
